# Relationships between chemical structure, mechanical properties and materials processing in nanopatterned organosilicate fins

**DOI:** 10.3762/bjnano.8.88

**Published:** 2017-04-13

**Authors:** Gheorghe Stan, Richard S Gates, Qichi Hu, Kevin Kjoller, Craig Prater, Kanwal Jit Singh, Ebony Mays, Sean W King

**Affiliations:** 1 Material Measurement Laboratory, National Institute of Standards and Technology, Gaithersburg, MD 20899, USA; 2 Anasys Instruments Incorporated, Santa Barbara, CA 93101, USA; 3 Components Research, Intel Corporation, Hillsboro OR 97124, USA; 4 Logic Technology Development, Intel Corporation, 5200 NE Elam Young Parkway, Hillsboro OR 97124, USA

**Keywords:** atomic force microscope, contact resonance, infrared spectroscopy, organosilicate, photothermal

## Abstract

The exploitation of nanoscale size effects to create new nanostructured materials necessitates the development of an understanding of relationships between molecular structure, physical properties and material processing at the nanoscale. Numerous metrologies capable of thermal, mechanical, and electrical characterization at the nanoscale have been demonstrated over the past two decades. However, the ability to perform nanoscale molecular/chemical structure characterization has only been recently demonstrated with the advent of atomic-force-microscopy-based infrared spectroscopy (AFM-IR) and related techniques. Therefore, we have combined measurements of chemical structures with AFM-IR and of mechanical properties with contact resonance AFM (CR-AFM) to investigate the fabrication of 20–500 nm wide fin structures in a nanoporous organosilicate material. We show that by combining these two techniques, one can clearly observe variations of chemical structure and mechanical properties that correlate with the fabrication process and the feature size of the organosilicate fins. Specifically, we have observed an inverse correlation between the concentration of terminal organic groups and the stiffness of nanopatterned organosilicate fins. The selective removal of the organic component during etching results in a stiffness increase and reinsertion via chemical silylation results in a stiffness decrease. Examination of this effect as a function of fin width indicates that the loss of terminal organic groups and stiffness increase occur primarily at the exposed surfaces of the fins over a length scale of 10–20 nm. While the observed structure–property relationships are specific to organosilicates, we believe the combined demonstration of AFM-IR with CR-AFM should pave the way for a similar nanoscale characterization of other materials where the understanding of such relationships is essential.

## Introduction

A fundamental objective of materials science and engineering is to understand, control, and exploit the relationships between the structure of a material at various length scales and its properties in order to create new materials with novel beneficial functionalities [1]. To date, the structure–property relationships of various materials have been understood at the macro- and microscale, and, in a few specific cases, even at the atomic-length scale [2]. The nanoscale characterization of structure–property relationships is experimentally elusive [3]. However, it has become a pervasive need for research currently focused on manipulating matter at nanometer length scales to take advantage of various nanoscale size effects [4–6]. It is a particularly pressing need for the semiconductor industry where the ability to resolve, map, and create nanoscale variations in chemical structure and materials properties will be needed to extend Moore’s law of transistor scaling into the single-digit nanometer regime [7–8]. Nanoscale structure–property characterization of grain boundaries and interboundary materials has also been recently identified as one of the current grand challenges in the research of ceramics [9]. Numerous other fields of science including chemistry, physics, and biology would additionally benefit from the ability to perform combined nanoscale characterization of chemical structure and materials properties, i.e., when there is a need to understand the structure–functionality relationships of sub-cellular components [10–14]. Interestingly, the lack of studies is not due to the absence of techniques to characterize properties at the nanoscale (there are many thanks to numerous advances in scanning probe microscopy (SPM) [15–21]). It is due to the non-existence of techniques to identify chemical structures with nanometer lateral (in-plane) resolution. Scatterometry and near-field optical microscopy (NSOM) techniques have allowed sub-wavelength features to be resolved [22–23]. However, the Abbe diffraction constraints prohibit direct spatial resolution of light-based spectroscopy techniques, such as infrared (IR) absorption spectroscopy, in the nanometer range [24]. Fortunately, these limitations have been recently overcome via combining optical spectroscopy with atomic force microscopy (AFM) and exploiting either near-field optical enhancements produced by the probe tip [25–26], or using the AFM probe to detect various photoinduced thermo-mechanical responses [24,27]. However, these techniques have yet to be routinely combined with other nanoscale SPM materials property measurement techniques to provide credible demonstrations of nanoscale structure–property characterization.

In this regard, we provide here a compelling demonstration of nanoscale chemical structure–mechanical property characterization by combining AFM-based IR (AFM-IR) [28] spectroscopy with contact resonance AFM (CR-AFM) [29] mechanical property measurements in the investigation of 20–500 nm wide fin structures fabricated in a nanoporous organosilicate thin film. Nanoporous organosilicates are of significant importance to the electronics industry for reducing various parasitic capacitances [30]. They are extremely susceptible to modifications of chemical structure and materials properties during processing because of the presence of labile organic components and a porous structure [31–32]. Using AFM-IR, we have recently been able to observe nanoscale variations in the loss of the organic component in an organosilicate induced by the plasma etching and ashing processes utilized to transfer lithographically defined features into these materials [32]. Likewise, we have also recently demonstrated the ability of CR-AFM to resolve nanoscale variations in the mechanical stiffness of organosilicate materials that have undergone similar but not identical types of processing [33–34]. By combining AFM-IR with CR-AFM in the investigation of the same material, we are now able to clearly demonstrate a correlation between the mechanical stiffness of the nanoporous organosilicate and the selective removal and reinsertion of terminal organic (CH_3_) groups in the matrix. Further examination of this effect as a function of fin width and additional recently demonstrated depth-profiling CR-AFM mechanical property measurements [34] indicate that the loss of CH_3_ groups and the increase of mechanical stiffness occur primarily at the exposed top and sidewall surfaces of the fins over a length scale of the order of tens of nanometers. These results provide a relevant demonstration of correlations between chemical structure, materials properties and processing at the nanoscale for other fields of nanoscale science [9–12].

## Experimental

### Fabrication of nanoporous fins

The nanoporous organosilicate fin structures examined in this study were fabricated using a previously described subtractive pitch quartering process [34–35]. Briefly, plasma-enhanced chemical vapor deposition (PECVD) was used to deposit a 120 nm thick organosilicate on a 100 nm SiO_2_ thin film previously grown on a 300 mm diameter Si(001) substrate. During deposition of the organosilicate, a second-phase organic pore building “porogen” material was co-incorporated in the organosilicate film and then sacrificially removed using UV curing to create a 33% nanoporous organosilicate material with a nominal Young’s modulus of 5 GPa [36]. The pitch quarter patterning process consisted of first depositing on the nanoporous organosilicate a quad-layer film stack consisting of a backbone layer, an anti-reflection coating, a second backbone layer and a hard mask. Standard 193 nm immersion lithography and etching techniques were utilized to form a grid pattern in the first backbone layer. A spacer dielectric was then deposited over the backbone grid and the backbone material was selectively removed. Standard plasma-etching techniques were then utilized to transfer this pattern into the second backbone layer that was subsequently coated with a second spacer material. The remaining second backbone material was then removed and the second spacer pattern transferred into the hard mask material and then into the nanoporous organosilicate using standard plasma-etching techniques. On completion of transferring the pitch quartered pattern into the nanoporous organosilicate, the remaining hard mask and plasma-etching residues were removed using standard plasma-ashing and wet chemical cleans.

**Figure 1:**
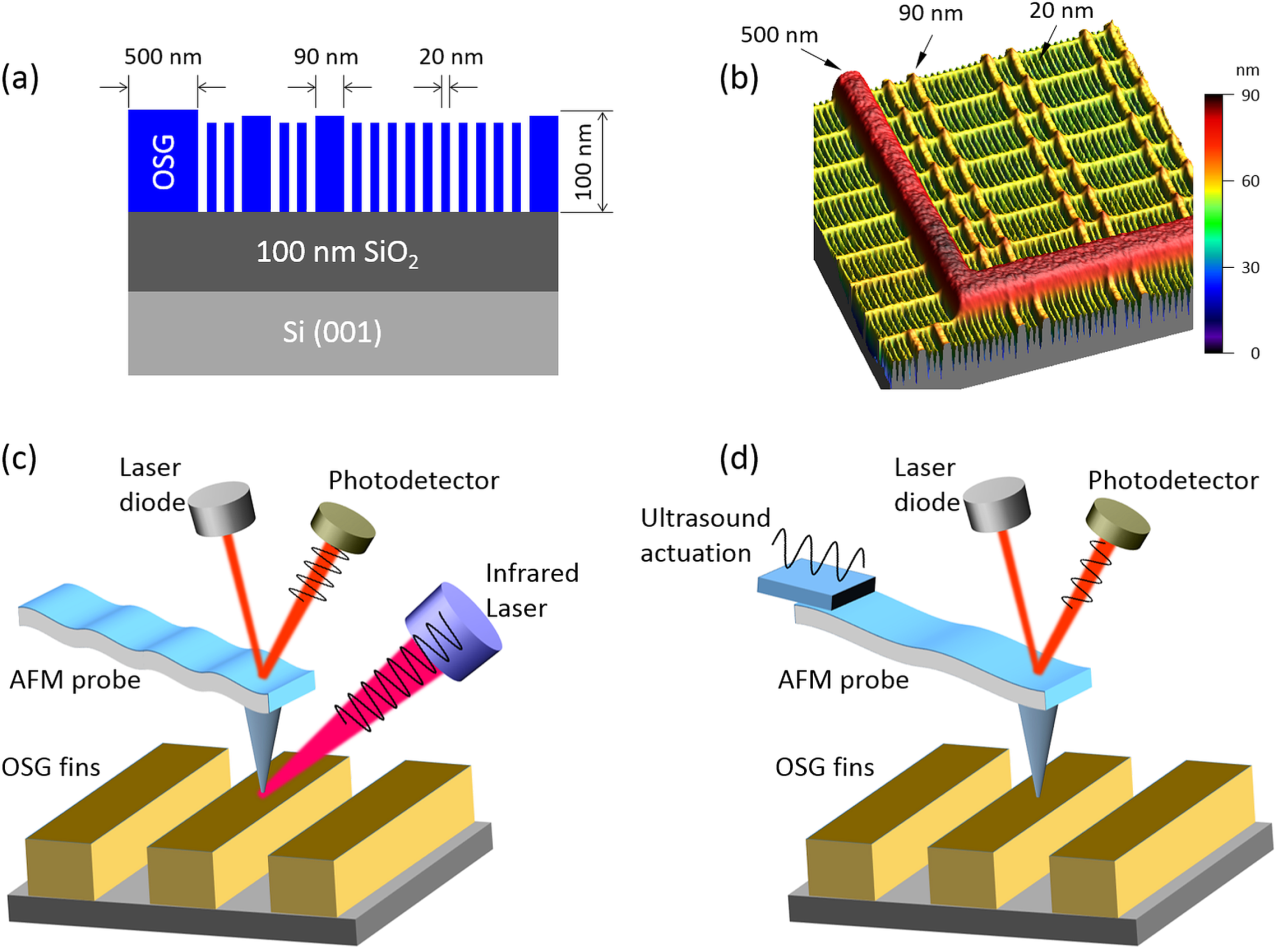
(a) Cross section schematic of the nanoporous organosilicate glass (OSG) fin structures investigated in this study. (b) AFM image (5 µm × 5 µm) of the surface topography of the actual structures investigated. (c) Schematic diagram of the AFM-IR measurements on the OSG fins. (d) Schematic diagram of the CR-AFM measurements on the OSG fins. Both (c) and (d) cartoons are drawn at relative scales to highlight the differences between the two techniques, with the impulse of the rapid sample expansion due to IR absorption causing a resonant oscillation of the AFM cantilever in AFM-IR (c) and the tip–sample contact mechanically vibrated at ultrasound frequencies in CR-AFM (d).

Figure 1 provides schematic illustrations and AFM images of the fin structures examined in this study. The schematic in Figure 1a specifically illustrates that an array of approximately 100 nm tall fins with widths of 20, 90, and 500 nm were fabricated in the organosilicate. As shown in the AFM image presented in Figure 1b, the length of the 20 nm wide fins was approximately 500 nm and these fins were nested within the wider 90 and 500 nm wide fins that run in length for more than 5 micrometers. It can be also observed that the height of the fins decreases by 5–10 nm with decreasing width due to pattern-dependent differences in the plasma etch/erosion rate of the photoresist and hardmask layers utilized to define and transfer the desired pattern into the nanoporous organosilicate [35]. As we will show, feature-size dependences also manifest as variations of composition and mechanical properties within the resulting nanoporous organosilicate fins.

After completion of fin formation, some samples were exposed to a standard silylation treatment in an attempt to restore some of the terminal organic groups removed from the nanoporous organosilicate by the patterning process. The details of the silylation treatment are described elsewhere [37–38]. Additional samples completed a metallization process flow that filled the voids between the organosilicate fins with Cu. This process consisted of the PVD of a TaN/Ta (TNT) barrier material followed by a Cu seed layer. The spaces between the fins were then completely filled with Cu via standard electroplating methods and a chemical mechanical polish was utilized to remove the protruding Cu material, leaving exposed the top of the nanoporous organosilicate fins as well as the TNT and Cu surfaces [39].

### AFM-IR, CR-AFM and FTIR characterizations

To follow changes in the chemical structure and mechanical properties of the nanoporous organosilicate induced by the pitch division patterning process as a function of feature size, we utilized the resonance enhanced mode of AFM-IR (Figure 1c) [32] and CR-AFM (Figure 1d) [34], respectively. In the resonance-enhanced AFM-IR technique, a tunable IR laser is pulsed onto the sample of interest and thermal heating of the sample occurs when the IR wavelength coincides with the IR vibrational absorption bands characteristic of the material [24,27]. The photoinduced heating causes a thermal expansion of the sample that is then detected as an oscillation in an AFM probe tip in contact with the sample. The repetition rate of the IR laser is tuned to a contact resonance of the AFM cantilever to maximize the oscillation amplitude of the cantilever. By sweeping the IR laser over the wavelengths of interest and monitoring changes in the amplitude of the AFM probe tip ringing, an IR absorption spectrum can be obtained that is directly analogous to traditional transmission IR techniques such as Fourier-transform infrared (FTIR) spectroscopy [28,32]. The spatial resolution for this technique has been previously demonstrated to be as low as 15–20 nm [40–41].

For CR-AFM measurements, the tip–sample contact is mechanically vibrated at various frequencies to detect the so called “contact resonance frequencies”. These CR-frequencies are characteristic of the tip–sample contact stiffness (higher frequencies for stiffer contacts) and depend on the mechanical properties of the tip and the sample, the contact geometry (e.g., spherical tip on a flat surface), and the applied force on contact [29]. By conducting CR-AFM measurements on different samples under the same conditions (same applied force and same contact geometry), differences between the mechanical properties (e.g., elastic modulus) of the samples are resolved. The spatial resolution of CR-AFM is determined by the contact radius established during measurements and it can be as small as 5–10 nm [33].

For the AFM-IR measurements presented here, a nanoIR2™ instrument was utilized. This instrument was equipped with a quantum cascade laser (QCL) and either an Arrow AFM probe with a force constant of 0.07–0.4 N/m (NanoandMore, Switzerland) or an Access-C AFM probe with a force constant between 0.06–0.9 N/m (AppNano, Mountain View, CA). The QCL was focused directly onto the sample at a polar angle of 30° and an approach angle of 60° from the front of the AFM probe, and swept continuously over the wavenumber range of interest [39]. The AFM-IR spectra were collected by tuning the repetition rate of the QCL to match a contact resonance of the AFM cantilever, typically the second flexural mode of the cantilever at ca. 180 kHz. This offers improved sensitivity over the OPO IR laser source used in previous investigations [32] and thus provides a better signal-to-noise ratio for the low thermal expansion of the organosilicate material. The oscillation amplitude of the probe at this frequency was then plotted versus the wavelength of the QCL to generate spectra. All experimental data were collected with the AFM probe in the contact mode using Analysis Studio software (Version 3.7, Anasys Instruments, Santa Barbara, CA) [37].

For comparison, transmission FTIR spectra were collected from the nanoporous organosilicate film before patterning using a Thermo Scientific Nicolet 6700 FTIR spectrometer and deuterated L-alanine doped triglycine sulfate (DLaTGS) detector. The spectra were acquired from 400–7000 cm^−1^ with 4 cm^−1^ resolution and the signal was averaged over 128 scans. The absorption spectrum of the Si substrate and thin-film substrate optical interference effects were removed using rigorous methods that accounted for the full-wave nature of light and have been previously described in detail [42–43].

For CR-AFM, single-point measurements were performed on the unpatterned blanket organosilicate film and the 500 and 90 nm patterned fins by mechanically vibrating the tip–sample contact from the base of the cantilever [34]. The AFM probe used for these measurements was a SEIH PPP probe (NanoSensors, Neuchatel, Switzerland) with the free (out of contact) first two eigenmode frequencies at 102.7 kHz and 642.6 kHz, respectively. The spring constant of the cantilever was determined to be 7.35 ± 0.05 N/m by a laser Doppler vibrometer. A lock-in amplifier with an internal signal generator (Signal Recovery AMETEK, Oak Ridge, TN) was used to vibrate the AFM cantilever and to detect the AFM photodiode signal (MultiMode 8, Bruker, Santa Barbara, CA) at the oscillation frequency. For each measurement, the tip was brought into contact at a setpoint of 60 nN applied force and the frequency of the imposed modulation was swept from 100 kHz to 1 MHz with a step of 250 Hz [33–34]. The elastic modulus calculations were made by considering the blanket film as a reference with a modulus of 3.5 GPa and reporting all the measured values of contact stiffness to the contact stiffness measured on the blanket film. The tip–sample contacts were treated as Hertz contacts [33].

## Results and Discussion

**Figure 2:**
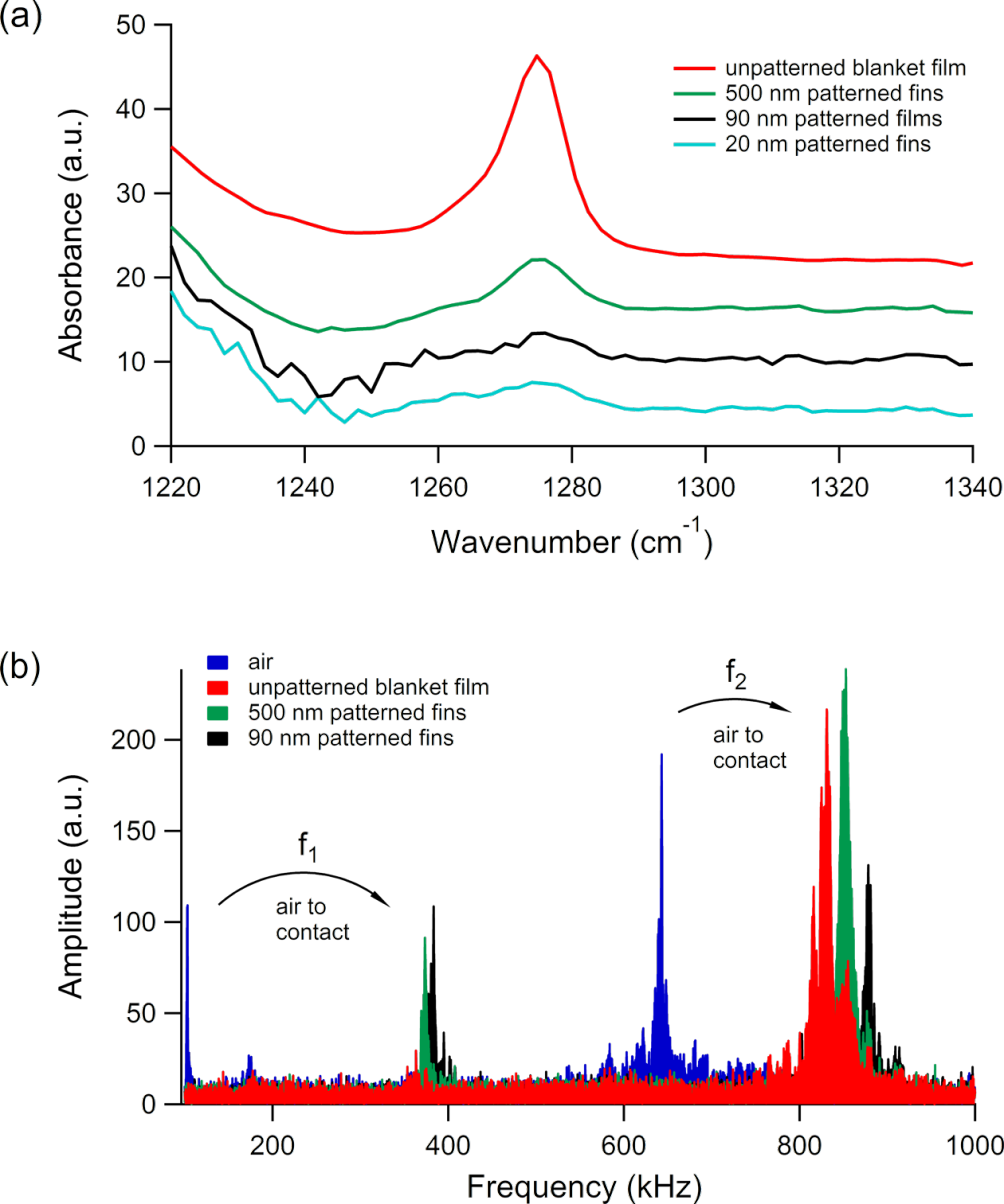
(a) Transmission FTIR and AFM-IR spectra of the symmetric SiC–H_3_ deformation mode from a nanoporous organosilicate unpatterned film and patterned fins (widths of 500 nm, 90 nm, and 20 nm), respectively. (b) CR-AFM spectra at 60 nN applied force of the first two eigenmodes from the same unpatterned film, 500 nm fins, and 90 nm fins that were measured in (a). The arrows indicate the frequency shifts of the two eigenmodes from air to contact.

To monitor changes in the chemical structure of the organosilicate fins induced by the patterning process, AFM-IR was used to focus specifically on relative changes in absorbance for the symmetric SiC–H_3_ deformation mode at about 1270 cm^−1^. This correlates directly with the concentration of terminal CH_3_ groups present in the nanoporous organosilicate structure. As has been detailed by many others [44], the incorporation of terminal organic groups within a silicate material radically disrupts the Si–O–Si network bonding and forces a more open/lower density structure to be adopted along with the creation of nanopores of varying size and interconnectivity. Numerous additional blanket/unpatterned film studies have shown a direct correlation between the concentration of such terminal organic groups and porosity and mechanical properties [45–46]. Figure 2a presents AFM-IR spectra of the symmetric SiC–H_3_ deformation band collected from both the 20, 90, and 500 nm wide fins after completion of the pitch division patterning process. Also included in Figure 2a is a micrometer-scale resolution transmission FTIR spectrum collected from an unpatterned thin film of the same nanoporous organosilicate [34]. To better facilitate a direct comparison, the IR spectra in Figure 2a were all scaled/normalized to have the same absorbance for the Si–O–Si stretching band at 1050 cm^−1^ (not shown in order for the relative changes in the lower absorbance SiC–H_3_ deformation band to be more easily observed). As can be seen, the absorbance for the SiC–H_3_ deformation band decreases for the patterned porous organosilicate and the absorbance decreases further as the width of the fin decreases. For both the 20 and 90 nm wide fins, the SiC–H_3_ deformation band is almost below the AFM-IR detection limits.

The loss of terminal Si–CH_3_ groups is attributed to removal of terminal organic groups by both the plasma etching applied to transfer the photolithographically defined pattern and the plasma ashing and wet cleans applied to remove the hardmask and polymeric plasma-etching residues [32,46–48]. The correlation between the decrease in the SiC–H_3_ absorbance and fin width is attributed to the limited penetration depth and diffusion length for ions, radicals and other chemically active species present during plasma etching and ashing, and wet cleans through the overlying hard mask and the interconnected porosity in the organosilicate. More specifically, as the fin dimensions decrease, the penetration depth and diffusion length start to represent an increasingly larger percentage of the fin width. The nearly complete disappearance of the SiC–H_3_ deformation band for the 20 and 90 nm wide fins suggests that the penetration depth and diffusion lengths in this case are of the order of 10–100 nm. This length scale range is consistent with both prior experimental and theoretical investigations of oxygen-radical diffusion and CH_3_ depletion in blanket thin film/unpatterned nanoporous organosilicate dielectrics [32,49]. We also note that while the CH_3_ removal could likely be observed via transmission FTIR measurements of the same macrostructures, the feature-size dependence would not have been observed because of the diffraction-limited micrometer-scale resolution of this technique.

To complement the above spectroscopy of chemical structures, CR-AFM measurements were additionally performed on the same nanoporous fin structures to assess relative differences in mechanical properties [50]. In Figure 2b, CR frequency spectra are shown for the unpatterned blanket film and the 500 and 90 nm fins that were acquired under the same applied force of 60 nN. Specifically, it is shown how the first two eigenmode frequencies of the cantilever shift from air (out of contact) to contact on each of the samples, with the largest shift on the 90 nm fins. The increase in the CR frequency shifts indicates a clear stiffening of the patterned nanoporous organosilicate, which points to a width-dependence of the mechanical stiffness analogous to that of the IR absorbance. The direct relationship between the observed CR frequency shifts and the stiffness of the materials in Figure 2b is supported by the requirement of preserving the same contact geometry, namely spherical tip of the same radius on a flat surface, during measurements. This was verified by the consistent results obtained over three alternative rounds of 10 measurements of each material. However, the CR-AFM measurements on 20 nm wide fins were not included in this discussion because the edge compliances have a significant contribution in this case [42], and a direct comparison with the CR-AFM measurements on the wider fins would therefore be inconsistent.

**Figure 3:**
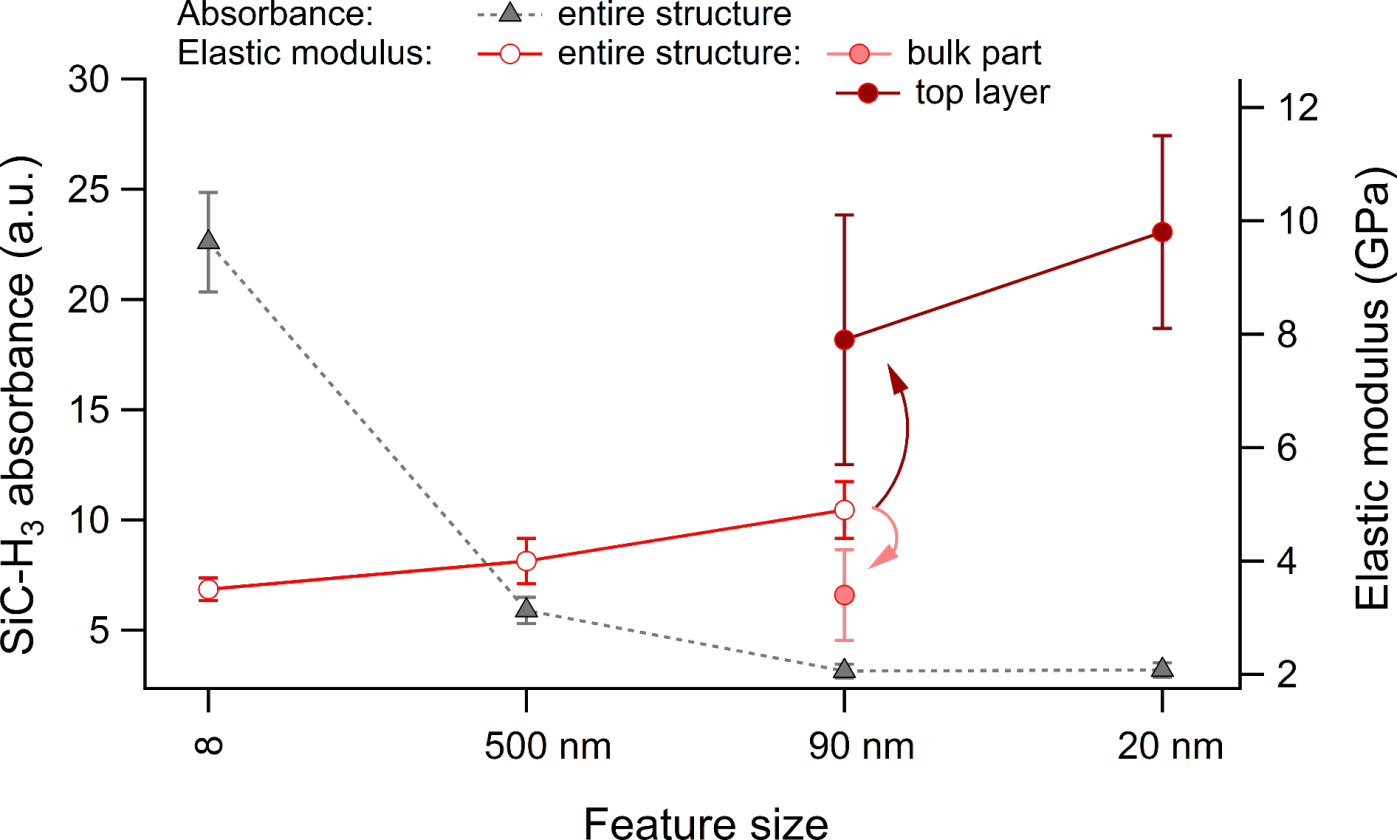
SiC–H_3_ absorbance (AFM-IR) and Young’s modulus (CR-AFM) as functions of the feature size for unpatterned and patterned nanoporous organosilicates. Note: the error bars for the AFM-IR SiC–H_3_ absorbance represents the maximum variability observed from spectra acquired at different sites with the same feature size. The details of the errors associated with the CR-AFM Young’s modulus measurements are covered in [34].

The decrease in SiC–H_3_ absorbance and increase in the elastic modulus as a function of fin width are summarized in Figure 3. In terms of elastic modulus, the 500 nm and 90 nm fins, when considered as homogeneous structures exhibit increases of 14% and 40%, respectively, with respect to the 3.5 GPa modulus of the unpatterned blanket film [34]. Both the increase in elastic modulus and decrease in SiC–H_3_ absorbance with the decrease in fin width suggest a conversion of the hybrid nanoporous organosilicate fins to a stiffer SiO*_x_* matrix [48]. In fact, additional depth-dependent CR-AFM measurements [42] and detailed modeling to include possible structural inhomogeneities and edge compliances have shown that about 20 nm of the top and sidewalls of the 90 nm fins consist of a thin “crust” layer with increased stiffness (*E* = 7.9 ± 2.2 GPa) relative to the remaining bottom portion of the fin that has mechanical properties closer to those of the unpatterned organosilicate (*E* = 3.4 ± 0.8 GPa). The analysis also showed that the volume of the 20 nm fins is almost entirely stiffened with a modulus of *E* = 9.8 ± 1.7 GPa (Figure 3). The elastic modulus values determined here for the stiffer regions of the fins are comparable with the elastic modulus determined in a previous study for the SiO*_x_* matrix of these porous organosilicate materials (*E* = 7.3 ± 0.2 GPa). It is therefore conclusive that the length scale for CH_3_ removal from the organosilicate by the patterning process is closer to the order of 10–20 nm and that fins of 20 nm or smaller will be almost completely depleted of CH_3_ during processing.

**Figure 4:**
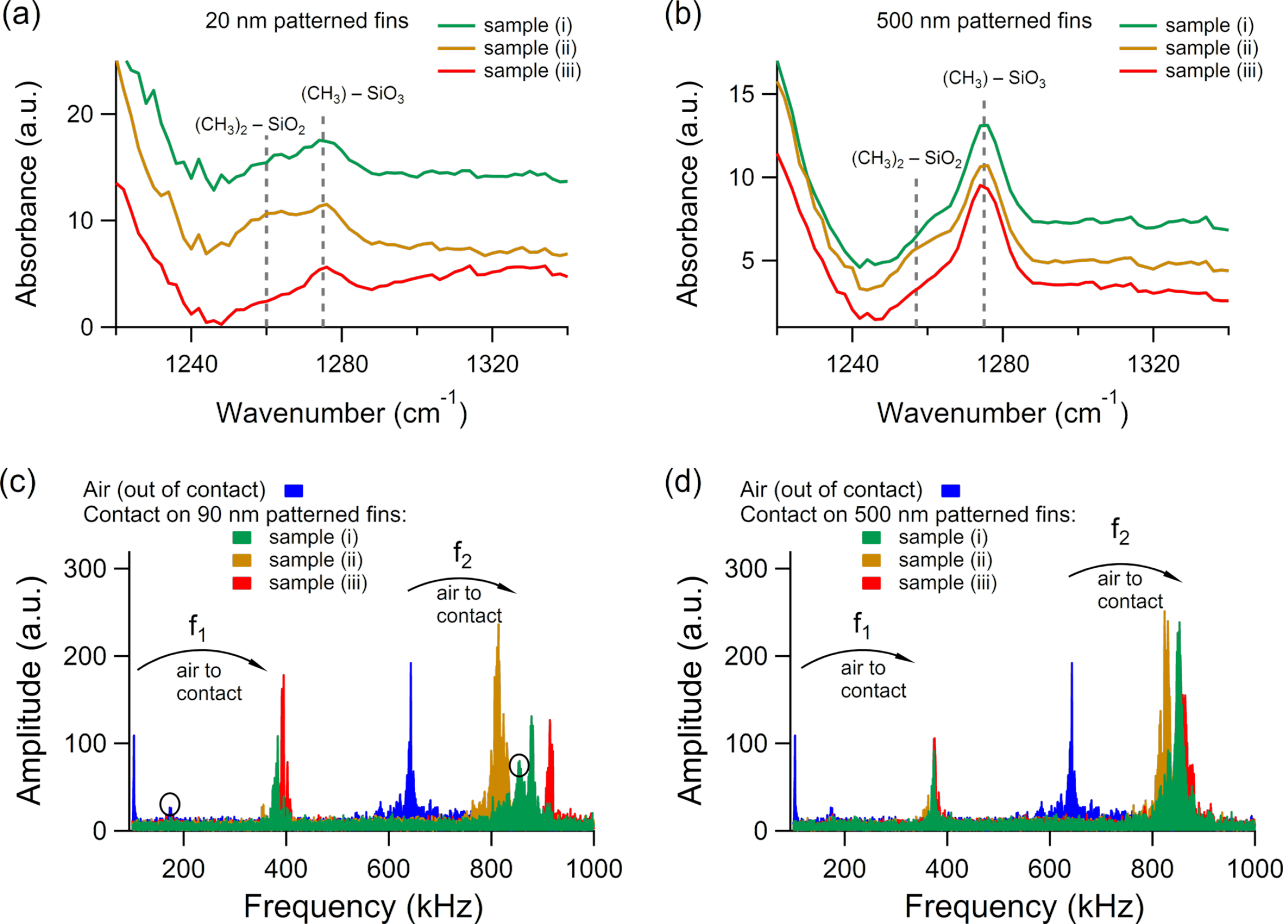
(a) and (b) AFM-IR spectra of the symmetric SiC–H_3_ deformation mode from the patterned nanoporous organosilicate fins with widths of 20 nm and 500 nm, respectively. (c) and (d) CR-AFM spectra of the first two eigenmodes from the patterned nanoporous organosilicate fins with widths of 90 nm and 500 nm, respectively. The circled peaks were observed in all the measurements and isolated as spurious resonances. The sample labeling is: (i) after pattern transfer, (ii) after chemical silylation treatment, and (iii) after metallization process flow.

While the removal of terminal organic groups from the matrix of the nanoporous organosilicate clearly results in a slight stiffening of the fins, it also results in the incorporation of significant amounts of hydroxyl groups (Si–OH) that can increase both the dielectric constant/capacitance and electrical leakage of nanoporous oganosilicate dielectrics [51]. This is typically counteracted by converting the hydroxyl groups back to terminal organic groups via a chemical silylation process that utilizes various alkoyxysilane or organosilazanes and takes advantage of the intrinsic porosity present to allow the silylating agent to easily penetrate the silicate matrix and react with hydroxyl groups [35]. To further examine the correlation between the concentration of terminal Si–CH_3_ groups and local mechanical properties, the previously examined fin structures were given such an industry-standard silylation treatment [35–36]. Figure 4 provides a comparison of the AFM-IR spectra from the 20 and 500 nm wide fins both before and after the silylation treatment. As shown in Figure 4a (curves i and ii), there was a clear overall increase in absorbance for the SiC–H_3_ deformation band after the silylation treatment for the 20 nm wide fins. In particular, the absorbance specifically increased at about 1265 cm^−1^. This is indicative of an increase in (CH_3_)_2_SiO_2_ entities as opposed to CH_3_SiO_3_ entities represented by an absorbance at 1275 cm^−1^ [44]. The increase in (CH_3_)_2_SiO_2_ entities after the silylation treatment is expected based on the structure of the silyation agent that includes two terminal CH_3_ groups [35–36].

In contrast, relatively little change was observed in the AFM-IR spectra of the 500 nm wide fins after the chemical silylation treatment. As shown in Figure 4b (curves i and ii), the CH_3_SiO_3_ absorbance at 1275 cm^−1^ remained almost unchanged and only a slight increase at 1265 cm^−1^ indicative of (CH_3_)_2_SiO_2_ entities was observed. This is consistent with the 500 nm fins exhibiting both reduced Si–CH_3_ loss and reduced stiffening induced by the patterning process relative to the 20 and 90 nm wide fins. Specifically, the reduced removal of terminal CH_3_ groups and the creation of a SiO*_x_* damage/crust layer presented fewer hydroxyl species for the silylation agent to react with.

To assess the impact of the silylation treatment on the mechanical properties of the organosilicate fin structures, CR-AFM measurements were again performed. These measurements interestingly showed a decrease in contact resonance frequency relative to the values before silylation. The stiffness decrease is more significant for the 90 nm fins (curves i and ii in Figure 4c) than for the 500 nm fins (curves i and ii in Figure 4d). The effect is visible on both CR eigenmodes and corresponds to a decrease in the elastic modulus of 37% for the 90 nm fins and 15% for the 500 nm fins with respect to the elastic moduli of the samples after pattern transfer (curves i in Figure 4c,d; in these calculations the fins were considered as homogeneous structures). This observation is fully consistent with the AFM-IR results showing a partial restoration of terminal Si–CH_3_ groups and is also consistent with the likely partial repair of the SiO*_x_* damage/crust layer created on the top surface of the fins by the patterning process. These results thus further confirm the correlation between terminal CH_3_ groups and the modulus of the nanoporous organosilicate.

To further strengthen the observed chemical structure–mechanical property relationship, the silylated nanoporous organosilicate fins were exposed to additional plasma and wet chemical processes to fill the spaces between the fins with Cu as in a typical state-of-the-art metal interconnect structure. This process flow specifically consisted of plasma cleaning, physical vapor deposition (PVD), chemical mechanical planarization (CMP) and wet chemical cleaning steps that have all shown the potential to remove terminal organic groups from the matrix of nanoporous organosilicate dielectrics in a similar fashion as observed previously by the pattern transfer process [31,52–53]. This is clearly shown in Figure 4a (curve iii) where the additional metallization processing also resulted in a decrease in SiC–H_3_ absorbance detected by AFM-IR from the 20 nm wide fin structures and that largely negated the effects of the silylation treatment. In contrast, the AFM-IR spectra from the 500 nm wide fin structures (curve iii in Figure 4b) remained again almost unchanged by the metallization process flow. This suggests a similar feature-size dependence of the removal of terminal CH_3_ groups. More importantly, CR-AFM measurements performed on the nanoporous organosilicate fins showed an increase in contact resonance frequency almost back to the values observed after the pattern transfer process (curves iii in Figure 4c,d), with an increase of about 25% for the 90 nm fins and 5% for the 500 nm fins with respect to the elastic moduli of the samples after pattern transfer (curves i in Figure 4c,d; in these calculations the fins were considered as homogeneous structures). This further confirms and strengthens the correlation between terminal CH_3_ groups and stiffness of nanoporous organosilicates.

## Conclusion

It has been demonstrated that AFM-IR and CR-AFM can be combined to perform chemical structure–mechanical properties characterization at the nanoscale. For the specific case examined here, a direct correlation has been observed between the removal and reinsertion of terminal organic groups and the elastic modulus for nanopatterned organosilicate fins. Specifically, an inverse correlation between concentration of terminal organic groups and elastic modulus was observed where selective removal of the organic component by plasma etching/ashing resulted in a stiffness increase and reinsertion via chemical silylation resulted in a stiffness decrease. Examination of this effect as a function of fin width further showed that the loss of terminal organic groups and stiffness increase occurred primarily at the exposed top and sidewall surfaces of the nanoporous organosilicate fins over a length scale of 10–20 nm. We believe these results provide a compelling case study for other material systems where the observation of similar nanoscale structure–property relationships may be essential in developing new materials or increasing understanding of nanoscale size effect phenomena.
